# Managingtomato bacterial wilt by suppressing *Ralstonia solanacearum* population in soil and enhancing host resistance through fungus-derived furoic acid compound

**DOI:** 10.3389/fpls.2022.1064797

**Published:** 2022-11-14

**Authors:** Meijin Ye, Hong Feng, Jianghong Hu, Qing Yu, Songqing Liu

**Affiliations:** Sichuan Provincial Key Laboratory for Development and Utilization of Characteristic Horticultural Biological Resources, College of Chemistry and Life Sciences, Chengdu Normal University, Chengdu, China

**Keywords:** antibacterial, soil-borne, plant disease, bio-control, natural product

## Abstract

Synthetic chemical pesticides are primarily used to manage plant pests and diseases, but their widespread and unregulated use has resulted in major health and environmental hazards. Using biocontrol microbes and their bioactive compounds is a safe and sustainable approach in plant protection. In this study, a furoic acid (FA) compound having strong antibacterial activity against soil-borne phytopathogenic bacterium *Ralstonia solanacearum* [causal agent of bacterial wilt (BW) disease] was isolated from *Aspergillus niger* and identified as 5-(hydroxymethyl)-2-furoic acid compound through spectroscopic analyses (liquid chromatography–mass spectrometry (MS), electron ionization MS, and NMR). The SEM study of bacterial cells indicated the severe morphological destructions by the FA compound. The FA was further evaluated to check its potential in enhancing host resistance and managing tomato BW disease in a greenhouse experiment and field tests. The results showed that FA significantly enhanced the expression of resistance-related genes (*PAL*, *LOX*, *PR1*, and *PR2*) in tomato and caused a significant reduction (11.2 log_10_ colony-forming units/g) of the *R. solanacearum* population in soil, resulting in the reduction of bacterial wilt disease severity on tomato plants and increase in plant length (58 ± 2.7 cm), plant biomass (28 ± 1.7 g), and root length (13 ± 1.2 cm). The findings of this study suggested that the fungus-derived FA compound can be a potential natural compound of biological source for the soil-borne BW disease in tomato.

## Introduction

Tomato is one of the most commonly consumed vegetables and dominant crops in the world. The cultivation and the production of tomato play a vital role in food security as well as socioeconomic development of people at the national and local levels (Asgarian et al., 2016; Moola et al., 2021). China plays an important part in tomato production, having the largest tomato cultivation area with the highest yield in the world. The annual tomato yield of China is 64.27 million tons, which is almost equal to one-third of the global tomato yield (Cen et al., 2022). Over 40 diseases have been reported to threaten the tomato crop in China, with 10 of them, including bacterial wilt, causing extensive damage across the country (Chiwaki et al., 2005). Bacterial wilt, which is of soil-borne nature, is a serious disease affecting the quality and the quantity of tomato especially in southern China. The yield losses in tomato caused by *Ralstonia solanacearum* vary from 0% to 90% depending on the cropping pattern, pathogen strain, cultivar, soil type, and climate (Nion and Toyota, 2015).

The bacterial wilt pathogen *R. solanacearum* is a soil-borne phytopathogen that affects both non-solanaceous and solanaceous plants (Chandrashekara et al., 2012; Khan et al., 2021). This is a motile, strictly aerobic, G^-^, and rod-shaped bacterium that has been reported to cause bacterial wilt disease in more than 180 plants of 45 plant families (Tahat and Sijam, 2010). In soil, the bacteria start an infection through the roots and severally affect the water transport by colonizing and blocking the xylem vessels, resulting in the development of wilting symptoms and signs including leaf yellowing, reduced growth, and death of the plants (Ahing and Wid, 2016; Iraboneye et al., 2021). This specific process of infection caused by *R. solanacearum* is known as vascular wilt disease and categorized as one of the most important bacterial diseases worldwide, causing severe losses to several economically important agricultural crops including tomato (Nion and Toyota, 2015).

Several studies have reported the difficulties of managing *R. solanacearum* because of its endogenous growth in plants, soil persistency in deeper layers, and water dispersal and its link with weeds (Nion and Toyota, 2015). Teli et al. (2018) also reported that management strategies such as crop rotation, field sanitation, and the use of disease-resistant cultivars have mostly failed to control *R. solanacearum*. Although synthetic chemicals have shown some effective results, they are linked with soil, underground water and air pollution, biodiversity loss, and residual toxicity impacts (Hassan and Chang, 2017; Fan et al., 2019; Malerba and Cerana, 2019). The adoption of alternative management techniques for BW management, such as the use of potentially effective biocontrol agents, has therefore gained more attention (Chamedjeu, 2018). However, there are issues with storage duration that come with using these microbes (Algam et al., 2010). In order to manage plant diseases, it is therefore necessary to search for alternative control measures, such as the use of naturally produced antimicrobial compounds or secondary metabolites (SMs) by microorganisms.

Among microbes, the fungus group has been reported to secrete a diversity of SMs; therefore, they have been considered a useful source to discover new biologically active compounds (Boustie and Grube, 2005). *Aspergillus* spp. are widely known for the production of chemically diverse SMs including cyclopentapeptide, pyranone, polyketide, and alkaloids that showed antiviral, anticancer, antioxidant, and antibacterial activities (Swathi et al., 2013; Holm et al., 2014; Ding et al., 2019). Several compounds isolated from *Aspergillus* spp. showed promising antibacterial activities. Diphenyl ether, isolated from *A. sydowii*, exhibited antibacterial activity against a range of bacteria (Liu et al., 2017). *Aspergillus* sp. produced an active compound, emodin, that showed antibacterial activity against *Bacillus subtilis* and *Staphylococcus aureus* (An et al., 2019).

This study was aimed to investigate the antibacterial compound produced by *Aspergillus* spp. against BW pathogen and evaluate it for the control of tomato BW disease. The most active antibacterial compound, identified as furoic acid (FA) compound, was found to have a destructive effect on bacterial cell morphology and caused an enhanced expression of disease resistance genes. FA was also active in decreasing the soil pathogen population and disease severity, thus resulting in improved plant growth.

## Materials and methods

### Microbes and culturing media

Previously identified fungus *A. niger* (obtained from College of Chemistry and Life Sciences, Chengdu Normal University, Chengdu, China) was used for the extraction of antibacterial compound. Potato dextrose agar and potato dextrose broth were used for culturing and for the seed broth preparation of *A. niger*. Solid-state fermentation (SFM) was used for *A. niger* fermentation. Beef extract peptone broth (BEPB) and agar medium were used for culturing *R. solanacearum* and for antimicrobial tests. The composition of each medium is presented in **Supplementary Table S1**. Bacterial wilt pathogen *R. solanacearum* (biovar 3, race 1) was cultured for its logarithmic phase on beef extract peptone solid medium at 30°C and 150 rpm/min for 16 h (Liu et al., 2016). The activated strain was grown to its logarithmic phase in 35 ml of BEPB under shaking incubation at 150 rpm/min and 30°C. The culture of the bacteria was centrifuged at 6,000 rpm, and by using sterilized distilled water the concentration of 10^8^ CFU/ml was maintained.

### Extraction and identification of antibacterial compound

**Figure 1:**
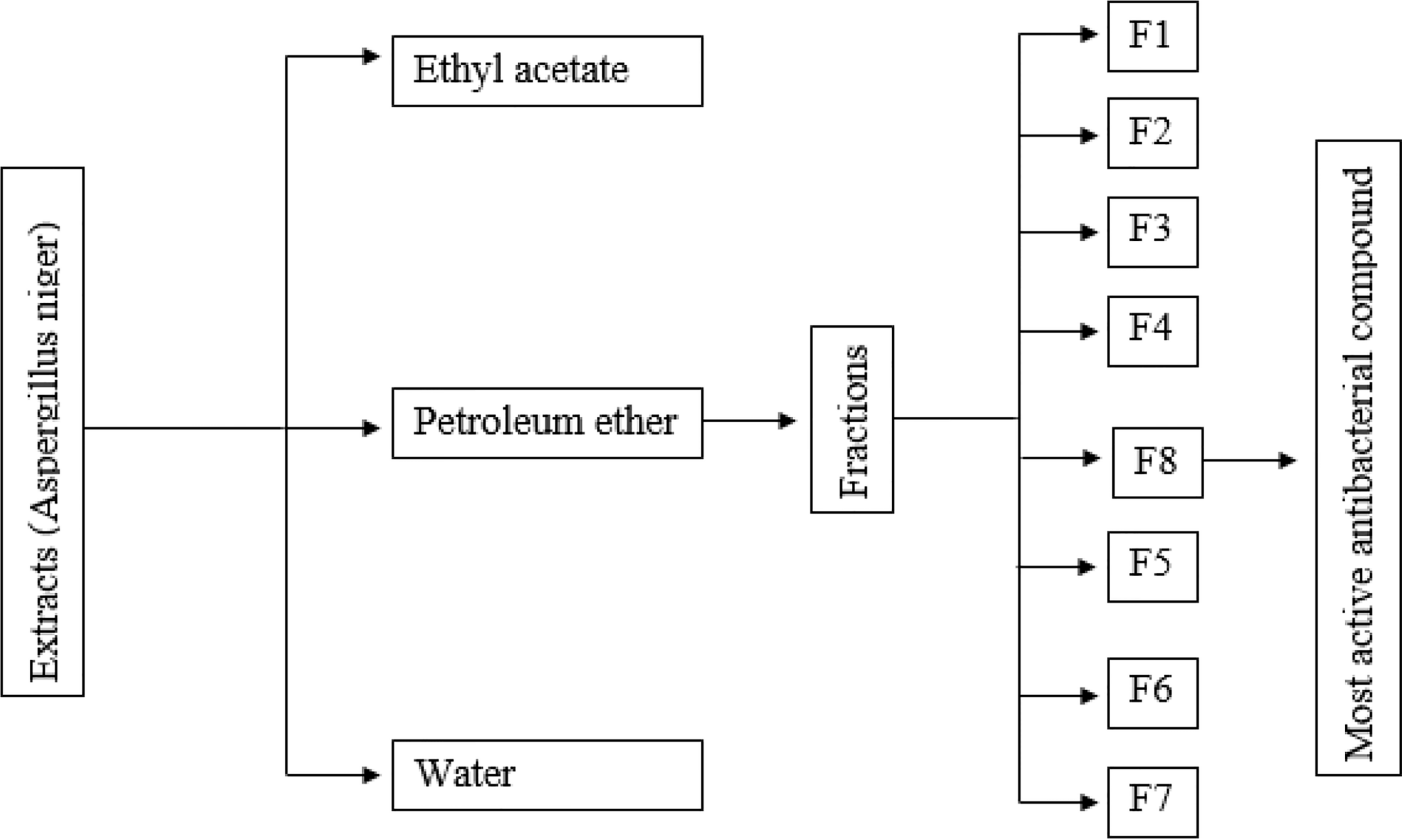
Schematic presentation of the isolation of the most active antibacterial compound.

Shallow plate fermentation method was used for fungal fermentation. SFM was inoculated with 15% (v/m) spore suspension of *A. niger* with 1 × 10^6^ spores/ml concentration and incubated for 1 week at 28°C. After the production of spores, the solid matrix was dried for 36 h and ground into particles. The spore powder of *A. niger* was collected, and the spore concentration was measured as 2 × 10^8^ spores/g. Crude ethanolic extract of spore powder was prepared by extracting spore powder four times for 4 h at 80°C using 10 L ethanol each time. In 300 ml of distilled water, 120 g of the extract was dissolved and then successively extracted four times with 300 ml of ethyl acetate (EA), petroleum ether (PE), and water to produce EA, PE, and water fractions. Separation of crude fractions of PE was done under the regulation of antimicrobial activity. Using silica gel column chromatography, the PE extract was fractionated, and the gradient was eluted. Thin-layer chromatography (TLC) analysis was used to evaluate the polarity composition of the fractions (200 ml), and those that have a similar composition were then concentrated and combined. The TLC analysis of the PE extract showed that it comprises different metabolites having various levels of bioactivity. Thus, additional fractionation was required to obtain more metabolites. Lastly, this separation procedure produced eight fractions (F1–F8) (**Figure 1**). The antimicrobial potential-guided separation of the most active fraction F8 was performed using reversed-phase silica gel column chromatography (CC), eluted with water–acetone (1:0, 9:1, 8:2, 7:3, 6:4, 5:5, 4:6, 3:7, 2:8, 1:9, and 0:1) to yield five sub-fractions (F8-1, F8-2, F8-3, F8-4, and F8-5). F8-1 (4.634 g) was again subjected to CC using silica gel (300–400 mesh) and eluted through petroleum ether–acetone (10:4). The further purification of sub-fraction was performed through silica gel CC with petroleum ether–acetone (5:2, v/v) as eluent to yield a white crystal compound (360 mg). The compound was subjected to liquid chromatography–mass spectrometry (MS), electron ionization MS, and NMR (^1^H-NMR and ^13^C-NMR) spectroscopic analysis, and by matching the obtained data with already published data, the structure of the compound was determined.

### Antibacterial activity

The crude extract and the fractions were tested for antibacterial activity against BW wilt pathogen *R. solanacearum*. At first, 100 mg/ml concentration of crude extract and active fractions were prepared separately, and then 10 μl of this concentration was poured to 1 ml of BEPB. Furthermore, this was inoculated with 100 μl *R. solanacearum* suspension [10^8^ colony-forming units (cfu)/ml] and incubated under shaking incubation at 28°C and 160 rpm to logarithmic phase. Dimethyl sulfoxide was kept as solvent control, and streptomycin was used as the positive control. Using a UV–vis spectrophotometer (Shenyang Ebetter Optics Co., Ltd.), the bacterial cell density was monitored at OD_λmax_ (420 nm). The bacterial inhibition rate I_R_ was calculated by using the following formula: I_R_ = (OD_0_ – OD_1_)/OD_0_ × 100. OD_0_ and OD_1_ represent the OD_λmax_ of treatment and blank control, respectively.

### Analysis of bacterial cell morphology

Destruction in bacterial cell morphology caused by the most active antibacterial compound (FA) was investigated through scanning electron microscopy. Bacterial cells under the treatment of FA and control (without treatment) were fixed using 2.5% glutaraldehyde and phosphate buffer for 1.5 h at 45°C, followed by washing with phosphate buffer (0.1 M, pH 7.2) for 5 min and fixing in osmium tetroxide (OsO_4_) for 1 h. Using phosphate buffer, the samples were washed, dehydrated in graded ethanol series (20%, 40%, 60%, 80%, and 90%) for 10 min each, and subjected to ethanol and CO_2_ to reach the critical point. By using gold ions, the samples were coated and subjected to scanning electron microscopy (SEM) evaluation (Kamonwannasit et al., 2013).

### Analysis of defense-related genes

Tomato plants (25 days old) were transplanted into sterilized culture medium pots (one plant per pot). Four treatments were maintained: T1, control plants without FA treatment and inoculation of pathogen (Ck); T2, plants inoculated with *R. solanacearum* suspension (10^8^ cfu/ml) (Rs); T3, plants treated with 9 ml FA suspension (300 μg/ml) (FA); and T4, plants inoculated with 15 ml of pathogen suspension (10^8^ cfu/ml) and treated with 9 ml FA suspension (300 μg/ml) (Rs+FA). According to the methods described by Kiefer et al. (2000), total RNA was isolated from the tomato plants. Briefly, 0.2 g of plant material was ground in liquid nitrogen to obtain tissue powder in which 1,000 μl TRIzol reagent was poured and mixed. The samples were incubated for 8 min on ice, followed by addition and mixing of 200 μl chloroform. The mixture was then centrifuged at 12,000 rpm for 12 min at 6°C. The obtained pellet was washed using ethanol (75%), mixed in 30 μl RNAse-free water, and stored at −80°C. The expression of defense-related genes (*PAL*, *LOX*, *PR1*, and *PR2*) was evaluated through real-time polymerase chain reaction (RT-PCR) using the isolated RNA from tomato plants under different treatments. The primers used for the analysis of defense-related genes are presented in **Supplementary Table S2**. For reference, Ubi3 was used. The RT-PCR reaction was conducted in 25 μl reaction mixture on DNA Engine Opticon2 Continuous Fluorescence Detection System. The composition of the reaction mixture and the operating conditions are given in **Supplementary Table S3**. RNAse-free water was used instead of cDNA for negative control. Following the extension stage, the fluorescence signal was evaluated immediately after 2-s incubation at 70°C, which prevents the possibility of primer dimer detection. The melting points of the PCR products were measured between 65°C and 95°C at the end of the cycles. Agar gel electrophoresis and melting curve analysis were used to confirm the specificity of the amplicons. The experiment was conducted in three replicates.

### Pot experiment

A pot experiment was conducted in a greenhouse for evaluating the potential of FA to manage the bacterial wilt of tomato. Plastic pots (20 cm in diameter) were filled with 1 kg soil (sand/clay/silt = 25%:25%:50%) each, and 20-day-old tomato plants (cultivar Rio Grande) were transplanted (one plant/pot). In each pot, 15 ml of pathogen suspension (10^8^ cfu) was added to initiate the bacterial wilt disease. After 3 days of inoculation with the pathogen, the rhizosphere of each plant was treated with 9 ml of sterilized water containing an increasing concentration of FA (control and 50, 100, 150, and 200 μg/ml). The treatments were applied through three holes surrounding the plant. Following horticultural recommendations, the plants were irrigated and fertilized for 50 days. Each treatment was applied in seven replicates using completely randomized design (CRD). The experiment was terminated after 50 days, and data were taken on the pathogen population in soil, disease severity, and plant growth parameters (plant length, root length, and plant biomass). Data on pathogen population and disease severity were converted to log_10_ and area under the disease progress curve (AUDPC) value, respectively, according to a previously described method (Khan et al., 2020). The experiment was repeated once, and the data of the two experiments were tested for significant difference and, in case of no significant difference, pooled for analysis.

### Field experiment

Field evaluation of FA compound for controlling BW disease in tomato was tested in an experimental field at the College of Chemistry and Life Sciences, Chengdu Normal University, Chengdu 611130, China, in March 2021 and repeated in March 2022. The field area was divided into three sets of blocks, and every set contained three blocks, each with a size of 6 m^2^. Tomato seedlings (20 days old) were transplanted in all blocks by maintaining two rows of tomato plants per block and eight plants in each row. The base of each plant in the two sets were treated with 100 ml of pathogen suspension (10^8^ cfu/ml) mixed with 300 ml of water. The plants in one of these two sets were treated with 20 ml of FA suspension (400 μg/ml) at the rhizosphere (T1: Rs+FA), while the other set was kept as untreated inoculated control (T2: Rs). The plants in the third set of blocks were kept as untreated and un-inoculated control (T3: control). The experiment was terminated after 50 days, and data were taken on soil bacterial population, disease severity, and plant growth parameters (plant height, root length, and plant fresh biomass). Data on disease severity were converted to AUDPC value according to a previously described method. Soil bacterial population was calculated twice: once at the start after 24 h of pathogen inoculation (initial: Pi) and once at the end of the experiment (final: Pf). The difference between Pi and Pf was calculated and expressed as decrease in soil bacterial population. The experiment was repeated once, and the data of the two experiments were tested for significant difference and, in case of no significant difference pooled for analysis.

### Statistical analysis

The experiments were conducted using CRD in the laboratory and greenhouse, while field tests were evaluated using randomized complete block design. The data were analyzed through Statistical Analysis System software version 8.0. Analysis was done using one-way analysis of variance. Tukey’s multiple-range test (*P* < 0.05) was used to evaluate the significant differences among treatment means.

## Results

### Antibacterial evaluation and identification of antibacterial compound

**Figure 2:**
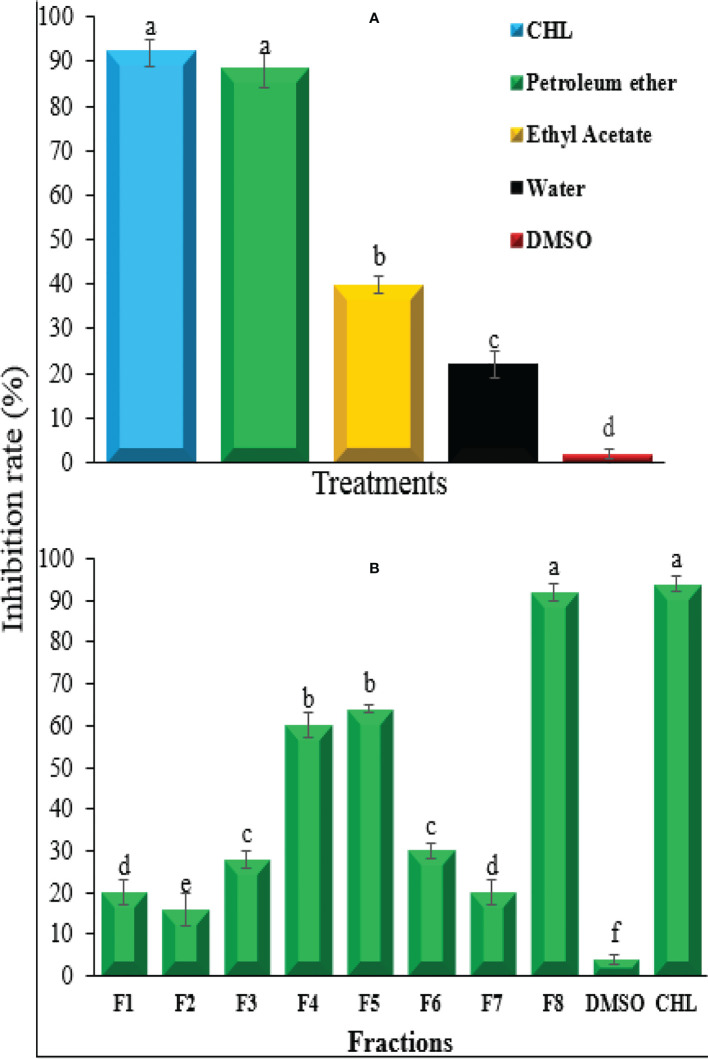
Growth inhibition rate of **(A)** niger extract using different solvents **(A)** and different fractions of petroleum ether extract **(B)** against bacterial wilt pathogen *Ralstonia solanacearum*. CHL, chloramphenicol; DMSO, dimethyl sulfoxide. Bars represent the standard error. Lower case lettering shows the significant difference among the treatments.

Different solvent extracts of *A. niger* were tested for the inhibition of *R. solanacearum* growth. Among different extracts, the petroleum ether extract showed the highest antibacterial activity that was equal to the positive control streptomycin. The inhibition rate of petroleum ether extract and streptomycin was 89.4% and 91.3%, respectively, followed by ethyl acetate extract and aqueous extract that showed 39.5% and 23.6% inhibition rate, respectively (**Figure 2A**). Based on the maximum inhibition rate, petroleum ether extract was selected for further fractionation and analysis of antibacterial compound. The fractionation of petroleum ether extract resulted in eight fractions showing varying degrees of inhibition rate against *R. solanacearum*. Among these eight fractions, F4, F5, and F8 showed a clearly higher antibacterial potential compared with others, and among these three fractions, significantly highest inhibition rate of 90.2% was shown by F8, which was similar to that obtained by positive control which indicated the presence of antibacterial compounds in these three fractions and especially in F8 (**Figure 2B**). As the fractions F4 and F5 showed inhibition rates of less than 60% and F8 only exhibited more than 90% inhibition rate, F8 was further processed for separation and purification of the antibacterial compound. Finally, a compound of white crystal nature was obtained. Based on their spectroscopic data, it was identified as FA compound—5-(hydroxymethyl)-2-furoic acid—with molecular formula C_6_H_6_O_4_ (**Supplementary Figures S1, S2**).

### Analysis of bacterial cell morphology

**Figure 3:**
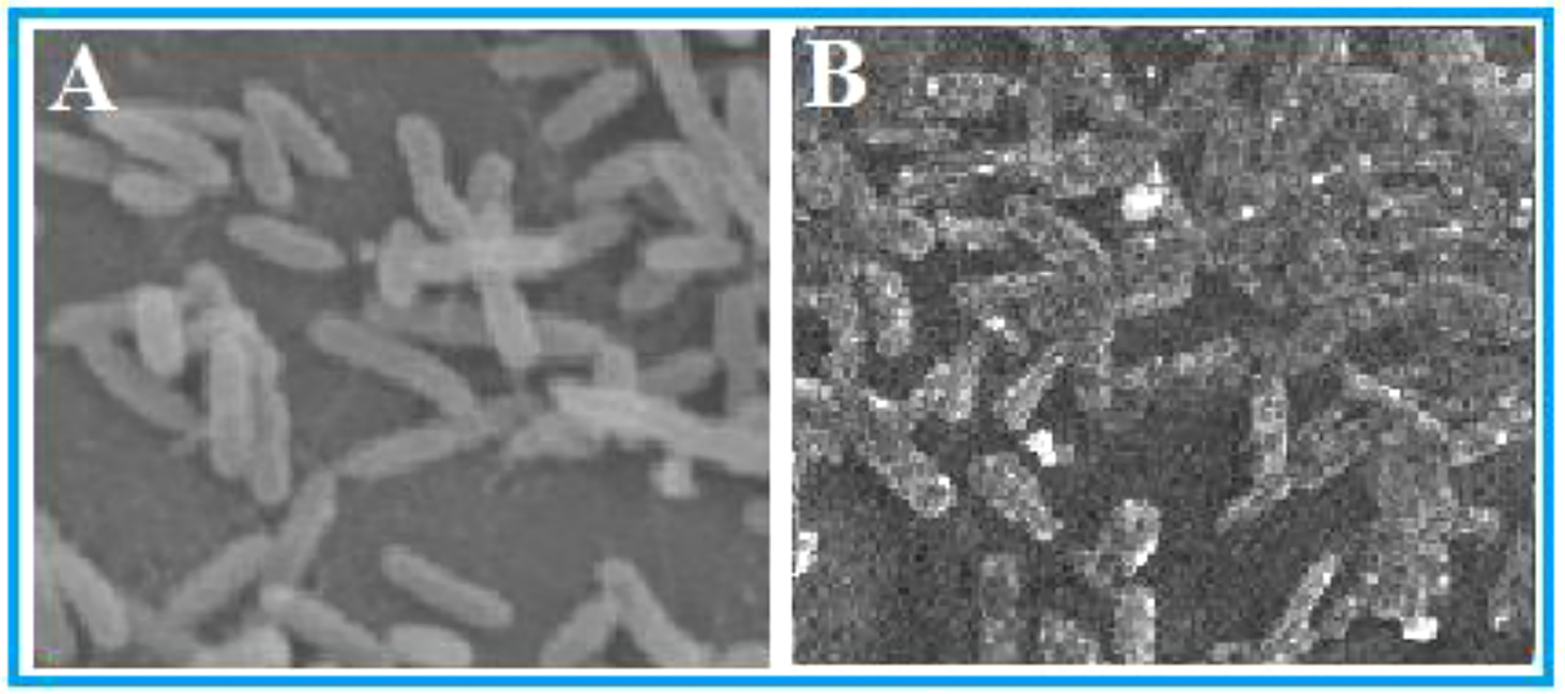
Morphological observations of *Ralstonia solanacearum* in SEM analysis. **(A)** Control: untreated bacterial cells and **(B)** cells treated with furoic acid compound.

Bacterial cell morphology under treatment of FA compound and without treatment was observed in SEM analysis. The micrographs clearly showed severe destructions in morphology of FA-treated bacterial cells. Compared with the control (**Figure 3A**), the FA-treated cells were swollen and the membranes were disrupted (**Figure 3B**). Leakage of cell content was obvious in the damaged cells, whereas in the untreated control the cells were in uniform morphology with a plain rod shape.

### Analysis of resistance genes

**Figure 4:**
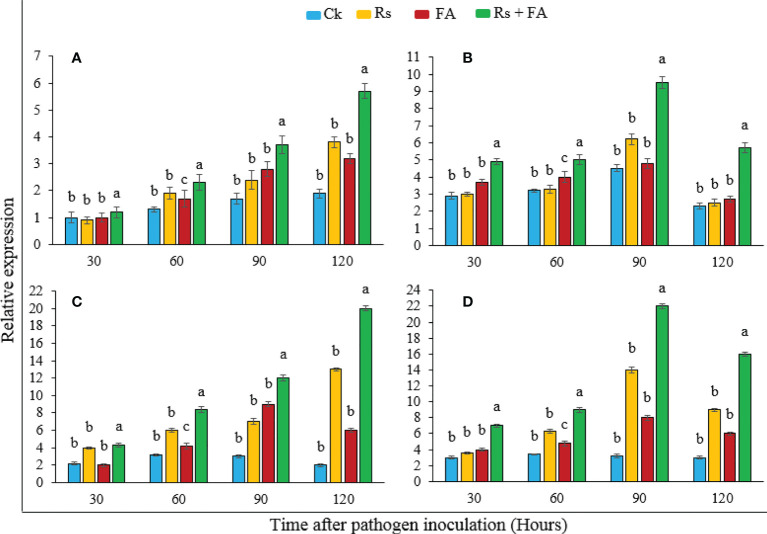
Relative expression of defense-related genes of tomato plants treated with furoic acid compound at different durations after pathogen inoculation. **(A)** *PAL*, **(B)** *LOX*, **(C)** *PR1*, and **(D)** *PR2*. Bars represent the standard error. Lower case lettering shows the significant difference among the treatments.

The expression of host defense-related genes (*PAL*, *LOX*, *PR1*, and *PR2*) in tomato plants was analyzed through RT-PCR after 30, 60, 90, and 120 h of pathogen inoculation under FA treatment (**Figure 4**). The results showed that the FA-treated pathogen-inoculated plants showed an induced expression of resistance genes (*PAL*, *LOX*, *PR1*, and *PR2*) more than the basal level of expression in other groups where the plants were treated with pathogen alone, FA only, or un-inoculated and untreated control pants (Ck). The induction of *PAL* and *PR1* genes was highest at 5.7- and 20-fold, respectively, at 120 h, while genes *LOX* and *PR2* showed a maximum expression of 9.5- and 22-fold at 90 h, respectively, although plants with only pathogen inoculation or treated only with FA also showed an induced expression of these genes as compared with the control, but at a significantly lower rate and fold than the pathogen-inoculated plants that were treated with FA.

### Greenhouse evaluation

#### Effect of FA compound on the growth of *R. solanacearum*-inoculated tomato plants

The potential of FA compound for controlling BW disease in tomato was investigated in a pot experiment. The results regarding plant growth parameters showed FA application to enhance plant growth (**Table 1**). Compared with untreated control plants, the plants treated with FA showed significantly higher plant length, root length, and plant biomass. The plant-growth-promoting effect of FA under bacterial wilt stress was dependent on its concentration. The lowest concentration of 50 μg/ml was not effective; however, at higher concentrations of 100, 150, and 200 μg/ml, FA application significantly enhanced the plant growth. The maximum plant length (64.4 ± 3.8 cm), biomass (74.4 ± 4.4 g), and root length (33.7 ± 2.3) were obtained by 200 μg/ml concentration, followed by statistically similar results obtained by 150 μg/ml concentration. Similar treatment effects were noticed in the repeated experiment (**Table 1**).

**Table 1:** Effect of different concentrations of FA on tomato plant growth inoculated with bacterial wilt pathogen in pot experiment.

Treatments (FA concentration, μg/ml)	Plant length (cm)	Biomass (g)	Root length (cm)
March 2021			
Control	26.4 ± 2.3 c	30.6 ± 1.8 d	11.1 ± 1.1 c
50	28.2 ± 2.7 c	30.3 ± 2.6 d	12.2 ± 1.6 c
100	42.7 ± 3.2 b	39.8 ± 2.9 c	18.2 ± 2.1 b
150	59.3 ± 4.2 a	68.3 ± 4.2 b	30.1 ± 2.8 a
200	61.4 ± 3.8 a	74.4 ± 4.4 a	33.7 ± 2.3 a
March 2022			
Control	20.4 ± 2.2 c	25.4 ± 2.2 c	8.8 ± 0.8 c
50	23.3 ± 2.5 c	27.4 ± 1.8 c	10.2 ± 1.0 c
100	46.7 ± 3.5 b	34.2 ± 2.8 b	14.3 ± 1.6 b
150	57.2 ± 3.2 a	62.6 ± 3.2 a	26.5 ± 2.0 a
200	60.6 ± 4.3 a	65.3 ± 3.5 a	28.3 ± 2.5 a

Each value is a mean ± SE. Means with the same letters in a column are significantly different according to Tukey’s multiple-range test (P < 0.05). The experiment was conducted twice: once in March 2021 and repeated in March 2022.

FA, furoic acid.

#### Effect of FA compound on AUDPC and soil suppression of *R. solanacearum* population

**Figure 5:**
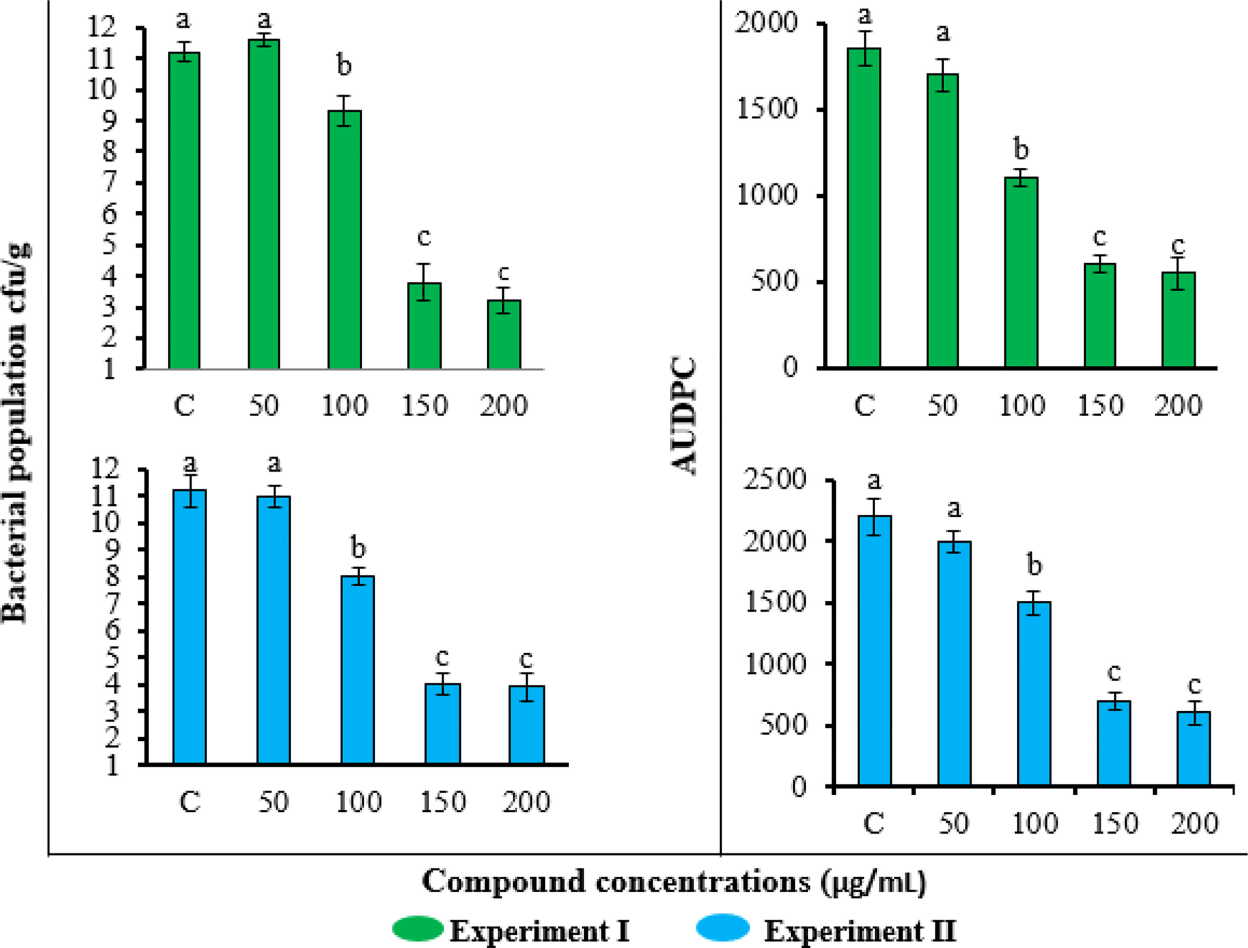
Effect of different concentrations of furoic acid on *Ralstonia solanacearum* population in soil and disease severity area under the disease progress curve value in pot experiment. C, control plants without any treatment. Bars represent the standard error. Lower case lettering shows the significant difference among the treatments.

The application of FA compound also reduced the bacterial wilt disease severity and pathogen population in soil (**Figure 5**). Consistent with the results of the plant growth parameters, the lowest concentration of 50 μg/ml was not effective in reducing disease severity and pathogen population, while the highest two concentrations of 150 and 200 μg/ml gave similar results. The application of FA at 200 μg/ml concentration showed the highest reduction in *R. solanacearum* population (3.1 log_10_ cfu/g) and the lowest AUDPC value (1850) followed by statistically similar results obtained by 150 μg/ml concentration. The treatments showed the same effects in the repeated experiment.

### Field evaluation

**Figure 6:**
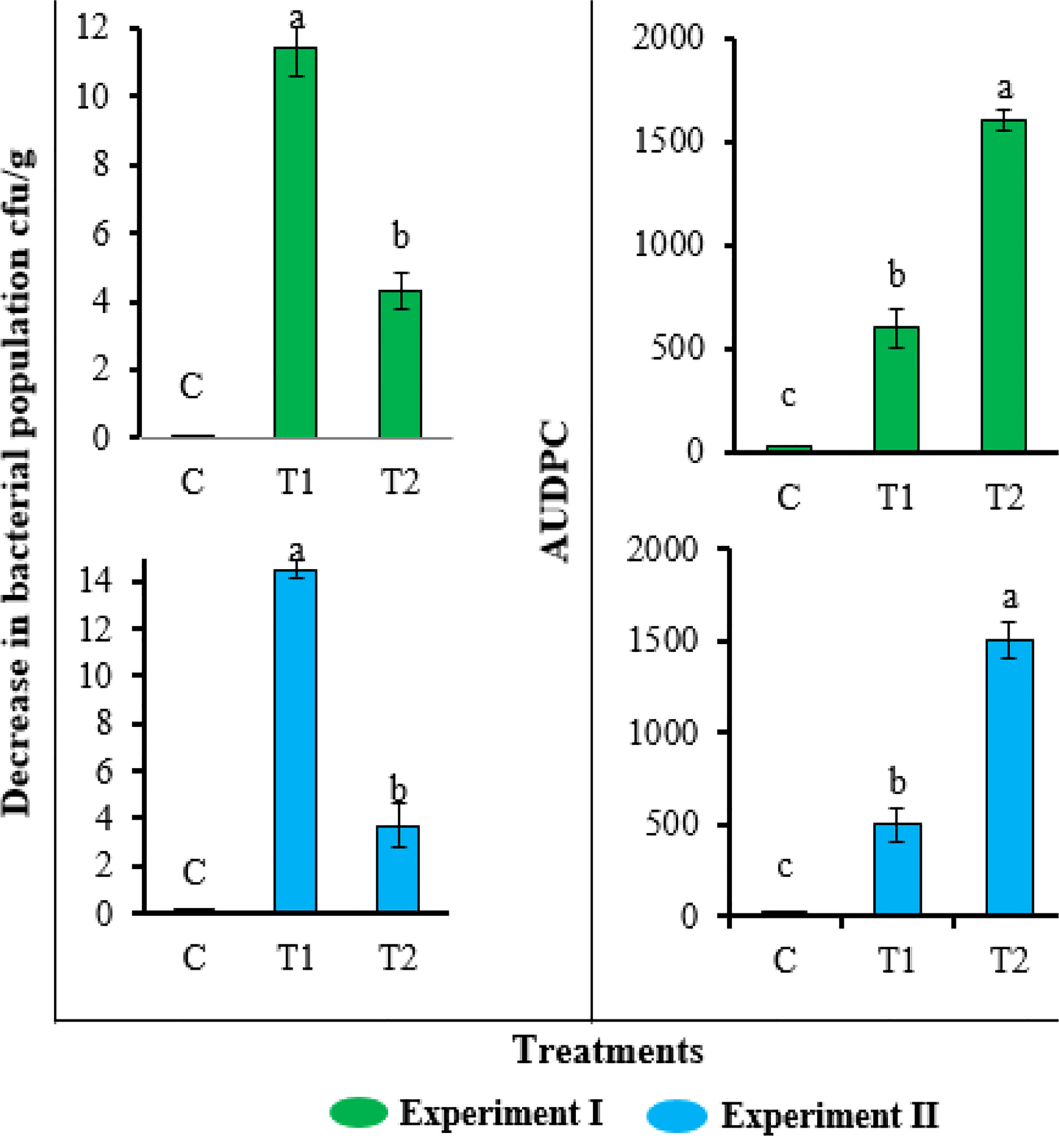
Effect of different concentrations of furoic acid (FA) on *Ralstonia solanacearum* population in soil and disease severity area under the disease progress curve value in field experiment. C, control plants; T1, plants inoculated with *R. solanacearum* and treated with FA (Rs + FA); T2, plants inoculated with *R. solanacearum* (Rs). Bars represent the standard error. Lower case lettering shows the significant difference among the treatments.

Consistent with the greenhouse results, the application of FA also reduced the bacterial wilt disease severity and soil population of *R. solanacearum* in field evaluation (**Figure 6**). The plant growth was also enhanced by the application of FA (**Table 2**). Compared with untreated inoculated control plants, the plants treated with FA exhibited a significantly lower AUDPC value (1,670) and the highest decrease (11.2 log_10_ cfu/g) in soil population of *R. solanacearum*. The inoculated plants under the treatment of FA had improved plant length (58 ± 2.7 cm), plant biomass (28 ± 1.7 g), and root length (13 ± 1.2 cm) as compared with the untreated inoculated plants. The results of the FA treatment showed a similar plant growth as that recorded for untreated and un-inoculated healthy control plants.

**Table 2:** Effect of different concentrations of FA on tomato plant growth inoculated with bacterial wilt pathogen in field experiment.

Treatments (compound concentration, μg/ml)	Plant length (cm)	Biomass (g)	Root length (cm)
March 2021			
Control	60 ± 3.6 a	69 ± 2.8 a	35 ± 2.3 a
Rs+FA	58 ± 2.7 a	70 ± 3.4 a	34 ± 1.8 a
Rs	26 ± 2.1 b	28 ± 1.7 b	13 ± 1.2 b
March 2022			
Control	52 ± 4.2 a	61 ± 3.2 a	28 ± 3.4 a
Rs+FA	54 ± 2.9 a	60 ± 2.6 a	30 ± 2.8 a
Rs	29 ± 1.8 b	32 ± 2.4 b	11 ± 1.4 b

Each value is a mean ± SE. Means with the same letters in a column are significantly different according to Tukey’s multiple-range test (P < 0.05). The experiment was conducted twice: once in March 2021 and repeated in March 2022.

Rs, Ralstonia solanacearum; FA, furoic acid.

## Discussion

Alternative plant disease and pest control methods are being developed all over the world to reduce the use of synthetic agricultural pesticides. This will save humans and protect the environment from the harmful effects of synthetic chemicals. The investigation and utilization of naturally produced microbial products is one way to do this. Previous studies have successfully shown that, by employing natural products from plants or microbes, they were able to reduce plant diseases. Among microbes, fungi have provided humanity with a variety of diverse bioactive compounds, thus becoming an effective group for research and development of new antimicrobial metabolites (Swathi et al., 2013). *Aspergillus* spp. has a variety of enzymes and produces several kinds of bioactive compounds. In this study, *A. niger* was investigated for the production of an antibacterial compound against *R. solanacearum*. Solvents of different polarities—water > ethyl acetate > petroleum ether—were used to extract the metabolites of *A. niger* spore powder. The metabolic extract obtained in petroleum ether demonstrated the highest antibacterial activity against *R. solanacearum*. Considering the presence of a main antibacterial compound in petroleum ether extract, it was further subjected to separation. Eight fractions, F1–F8, were obtained from petroleum ether extract, the most active fraction was analyzed, and FA compound was identified to have the highest antibacterial activity after spectroscopic analysis. The purified antibacterial FA compound was further evaluated for its effect on bacterial morphological destruction and the potential to manage the bacterial wilt of tomato in greenhouse and field conditions.

The results of the SEM analysis clearly demonstrated the membrane-damaging effect of FA compound against *R. solanacearum*. This compound is categorized as a furan ring derivative. Several studies reported the antimicrobial effects of furan ring derivatives against a range of pathogenic microbes such as *Bacillus megaterium*, *Alternaria alternate*, *Escherichia coli*, and *S. aureus* (Dai et al., 1989; Lewkowski, 2003; Zhang et al., 2019). The antibacterial effect could be ascribed to the enhanced permeability of the FA compound to the pathogen cell membranes, which causes membrane rupture and protein alterations. This eventually causes a disruption in cellular metabolism, which results in bacterial cell death (Premanathan et al., 2011; Vani et al., 2011; Akbar et al., 2020). The SEM results of our study also confirm this mechanism of antibacterial activity.

The results from plant experiments revealed that the application of FA caused a significantly enhanced expression of defense-related genes in tomato plants and suppressed the *R. solanacearum* population in the soil, resulting in the reduction of bacterial wilt disease severity and an increase in plant growth. Plant defiance can be improved through the use of exogenous elicitors in the form of plant- or microbe-based natural compounds. Fungal microbes have a large number of natural antimicrobial compounds that may act as natural elicitors, which act as host resistance inducer to enhance plant defense against pathogens. Several antimicrobial compounds such as aspyrone, γ-dehydrocurvularin, and penipratynolene were isolated previously from *Aspergillus* fungi against soil-borne pathogens (Kimura et al., 1996; Kusano et al., 2003). Kimura et al. (2007) isolated 5-hydroxymethyl-2-furoic acid from *Aspergillus* sp. and reported its antimicrobial activities against the root knot nematode. They also reported the improved plant growth of several crops when 5-hydroxymethyl-2-furoic acid was applied.

The large volume of the antimicrobial compound required for field broadcasting can be reduced by applying it selectively to the plant rhizosphere. In seed-bed soils for disease-free seedlings and for small-scale agricultural systems like those used in many developing countries, this selective treatment can be easily practiced. The use of this furoic acid compound derived from *Aspergillus* fungus has a high potential for consideration as an active integrated disease management component of BW management. As a natural product, it is environment-friendly, and it is difficult for pathogens to resist it. This compound can be used in combination with other IDM strategies to enhance the management of the bacterial population in the soil—for example, BW pathogen is killed by soil temperatures of 45°C or above for about 2 days (Kangkiattikajorn et al., 2007). The soil application of this compound with plastic mulching during scorching sunny days before tomato transplantation could effectively reduce the bacterial population in soil.

## Conclusion

The findings of this study revealed the strong antibacterial potential of furoic acid compound derived from *A. niger* against soil-borne bacterium *R. solanacearum*, the causal agent of bacterial wilt disease. The compound caused severe morphological destructions to *R. solanacearum*. The application of this compound to the pathogen-infested soil transplanted with tomato plants enhanced the resistance of the tomato plants by increasing their expression of defense-related genes. This compound, when applied to the soil, effectively reduced the soil population of *R. solanacearum* and decreased the bacterial wilt disease severity, resulting in the improvement of plant growth. The current study suggests that the furoic acid compound has potential applications in the management of bacterial wilt disease in tomato and possibly in other crops.

## Data availability statement

The original contributions presented in the study are included in the article/**Supplementary Material**. Further inquiries can be directed to the corresponding author.

## Author contributions

MY and SL: conceptualization, writing, review, editing, and supervision. MY and SL: writing—original draft and funding acquisition. JH and QY: methodology. MY, HF, and JH: investigation. QY: resources. All authors contributed to the article and approved the submitted version.

## Funding

This work was sponsored in part by the Innovation and Entrepreneurship Training Program for College Students 202214389015, 202214389038, S202214389106, S202114389064 Chengdu Normal University Key Project(CS19ZA08), and Special Research Project of Chengdu Normal University(ZZBS2019-05).

## Conflict of interest

The authors declare that the research was conducted in the absence of any commercial or financial relationships that could be construed as a potential conflict of interest.

## Publisher’s note

All claims expressed in this article are solely those of the authors and do not necessarily represent those of their affiliated organizations, or those of the publisher, the editors and the reviewers. Any product that may be evaluated in this article, or claim that may be made by its manufacturer, is not guaranteed or endorsed by the publisher.
