# Supplementary material for: Managingtomato bacterial wilt by suppressing Ralstonia solanacearum population in soil and enhancing host resistance through fungus-derived furoic acid compound
Source: Front Plant Sci. 2022 Nov 14;13:1064797. doi: 10.3389/fpls.2022.1064797 (PMC9703000; doi:10.3389/fpls.2022.1064797)
Supplement: Supplementary file 1 [file DataSheet_1.doc]

Table S1: Beef extract peptone liquid medium

| **Media** | **Ingredient** | **Quantity (g/Litter)** |
| --- | --- | --- |
| **Beef extract peptone liquid medium** | Peptone | 10 |
|  | NaCl | 5 |
|  | Beef extract | 3 |
| **Beef extract peptone solid medium** | Peptone | 10 |
|  | NaCl | 5 |
|  | Beef extract | 3 |
|  | Agar | 20 |
| **Potato dextrose agar** | Potato | 200 |
|  | Glucose | 20 |
|  | Agar | 20 |
| **Solid-state fermentation medium** | NaNO_3_ | 1 |
|  | Sucrose | 131 |
|  | Wheat bran | 8 |

Table S2. Primers used for the expression analysis of defense related genes

| **Gene** | **Primer sequence (5'-3')** | | **Accession No.** |
| --- | --- | --- | --- |
| *LOX* | F | ATCTCCCAAGTGAAACACCACA | U13681 |
|  | R | TCATAAACCCTGTCCCATTCTTC |  |
| *PAL* | F | CTGGGGAAGCTTTTCAGAATC | AW035278 |
|  | R | TGCTGCAAGTTACAAATCCAGAG |  |
| *PR1* | F | GCCAAGCTATAACTACGCTACCAAC | DQ159948 |
|  | R | GCAAGAAATGAACCACCATCC |  |
| *PR2* | F | GGACACCCTTCCGCTACTCTT | M80604 |
|  | R | TGTTCCTGCCCCTCCTTTC |  |
| *Ubi* | F | TCGTAAGGAGTGCCCTAATGCTGA | X58253 |
|  | R | CAATCGCCTCCAGCCTTGTTGTAA |  |

Table S3: Composition and operating conditions of real-time PCR used in expression analysis of defense related genes.

| **Composition** | |
| --- | --- |
| **Substance** | **Amount** |
| SYBR green master mix | 12.5 μl |
| GFP-qp-F | 0.2 μL |
| GFP-qp-R | 0.2 μL |
| cDNA | 1 μL |
| RNase-free water | Remaining volume up to 25 μL |
| **Operating conditions** | |
| denaturation at 95 °C | 3 min |
| 35 cycles at 95 °C | 20 s |
| Annealing | 20 s |
| Extension at 95 °C | 20 s |


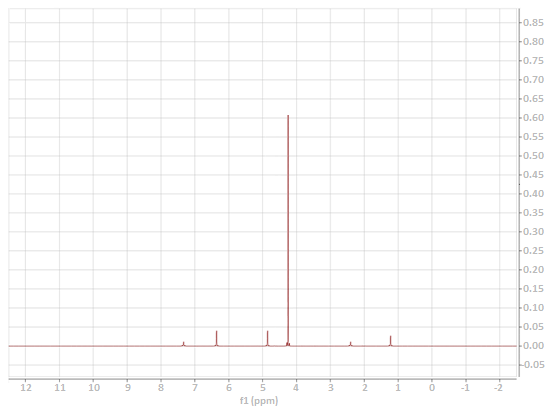


**Figure S1**. H-NMR spectrum of 5-(hydroxymethyl)-2-furoic acid compound.


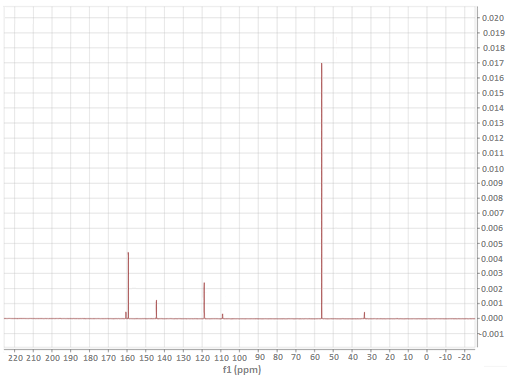


**Figure S2**. C-NMR spectrum of 5-(hydroxymethyl)-2-furoic acid compound
